# Bear bile powder ameliorates type 2 diabetes *via* modulation of metabolic profiles, gut microbiota, and metabolites

**DOI:** 10.3389/fphar.2022.1090955

**Published:** 2023-01-04

**Authors:** Xing-Ling Chen, Ke Cai, Wen Zhang, Shu-Lan Su, Li-Hui Zhao, Li-Ping Qiu, Jin-Ao Duan

**Affiliations:** ^1^ Jiangsu Key Laboratory for High Technology Research of TCM Formulae, National and Local Collaborative Engineering Center of Chinese Medicinal Resources Industrialization and Formulae Innovative Medicine, Jiangsu Collaborative Innovation Center of Chinese Medicinal Resources Industrialization, Nanjing University of Chinese Medicine, Nanjing, China; ^2^ Fujian Guizhentang Pharmaceutical Co., Ltd., Huian, China

**Keywords:** type 2 diabetes mellitus, bear bile powder, metabolomics, intestinal microbiota, short-chain fatty acid

## Abstract

**Introduction:** Bear bile powder (BBP) is widely used in the clinic and has a hypoglycemic effect, but its mechanism is not clear.

**Methods:** In this study, type 2 diabetes mellitus (T2DM) rats induced by a high-sugar and high-fat diet combined with streptozotocin were given BBP, and biochemical indexes, pathological sections, metabonomics, intestinal microbiota (IM) and short-chain fatty acids (SCFAs) were determined.

**Results:** The results showed that BBP could reduce blood glucose, relieve inflammation, insulin resistance, and lipid metabolism disorder, and alleviate tissue damage of the liver, spleen, kidney, and pancreas in T2DM rats. It is worth noting that BBP can reverse the changes in blood and urine metabolites in T2DM rats, which are mainly related to tryptophan metabolism, pentose and glucuronate interconversions, starch and sucrose metabolism, and glycerophospholipid metabolism. In addition, BBP restored IM disorder in T2DM rats, decreased the abundance of *Allobaculum*, *Blautia*, *Dubosiella*, and *Anaerostipes*, enriched the abundance of *Lactobacillus*, *Romboutsia*, *UCG-005*, and *norank_f__Eggerthellaceae*, and increased the concentration of SCFAs in intestinal contents.

**Discussion:** These findings suggest that BBP may improve T2DM by regulating multiple metabolic pathways, IM composition, and SCFAs levels.

## 1 Introduction

Type 2 diabetes mellitus (T2DM) is a metabolic disease caused by a relative deficiency of insulin secretion or insulin resistance (IR) (Daryabor et al., 2020). Uncontrolled diabetes can lead to macrovascular diseases, such as coronary heart disease, stroke, and peripheral arterial disease, as well as microvascular diseases, including complications such as diabetic nephropathy, retinopathy, and peripheral neuropathy (Tomic et al., 2022), seriously affect human health and quality of life. According to the statistics of the International Diabetes Federation in 2021, there are about 537 million people with diabetes worldwide, and the number is expected to rise to 783 million in 2045, of which T2DM accounts for more than 90% of all cases (International Diabetes Federation, 2021). Most western medicines not only play the role of anti-T2DM but also produce a lot of adverse reactions (Lee et al., 2021). The treatment of diabetes with traditional Chinese medicine has the characteristics of multi-components, multi-targets, and few side effects, so it has attracted more and more attention.

Bear bile powder (BBP) is dry bile collected from black bears (*Selenarctos Thibetanus*) or brown bears (*Ursus Arctos*) (Cai et al., 2022). BBP has been used in traditional Chinese medicine for thousands of years, with the effect of clearing heat, calming the liver, and clearing vision (Wang et al., 2016). Modern pharmacological studies have shown that BBP has the effects of protecting the liver and gallbladder, protecting the kidney, treating ophthalmopathy, regulating intestinal function, anti-inflammation, regulating blood sugar, reducing blood lipids, and so on (Li X. Y et al., 2022). The main chemical constituents of BBP include chenodeoxycholic acid (CDCA), ursodeoxycholic acid (UDCA), taurochenodeoxycholic acid (TCDCA), tauroursodeoxycholic acid (TUDCA), and so on. TUDCA can alleviate the disorder of lipid metabolism, reduce oxidative stress and intestinal inflammation, and improve blood glucose homeostasis and insulin sensitivity (Zhang et al., 2022). UDCA has a direct protective effect on pancreatic *β*-cells and can alleviate the expression of endoplasmic reticulum stress and pro-inflammatory response in diabetes (Chung et al., 2016; Mooranian et al., 2020). CDCA can increase the insulin sensitivity of skeletal muscle, which leads to an increase in systemic insulin sensitivity (Zhong et al., 2022). However, the mechanism of BBP in treating T2D isn't clear.

Metabonomics can comprehensively analyze the changes of endogenous metabolites under exogenous stimulation and provide the possibility for exploring the mechanism of disease occurrence and drug treatment (Pereira et al., 2022). The characteristic of the overall interaction of metabonomics is in line with the theory of multi-pathway action of traditional Chinese medicine. Intestinal microbiota (IM) plays an important role in controlling host physiology and metabolism (Fan and Pedersen, 2021). The imbalance of IM may lead to the progression of some diseases, including metabolic diseases, cardiovascular diseases, autoimmune or inflammatory diseases, etc (Schupack et al., 2022). A large number of studies have shown that T2DM is closely related to the abnormal composition and/or function of IM.

Therefore, in this study, UPLC-Q-TOF/MS was used to detect plasma and urine of rats, 16SrDNA high-throughput sequencing was used to analyze the composition of IM in rat cecal contents, and GC-FID was used to detect short-chain fatty acids (SCFAs). In addition, the interaction between IM and host indexes of T2DM rats treated with BBP was analyzed. To explore the antidiabetic mechanism of BBP on T2DM rats induced by a high-sugar and high-fat diet combined with streptozotocin (STZ). These findings may provide a promising strategy for the treatment of T2DM.

## 2 Materials and methods

### 2.1 Materials and chemicals

BBP (No. 20210601, Fujian Guizhentang Pharmaceutical Co., Ltd., China), STZ (No. WXBD7077V, Sigma, United States), sodium citrate buffer (No. 20210615, Beijing Solebao Technology Co., Ltd., China), metformin (No. ABZ8820, Sino-US Shanghai Squibb Pharmaceuticals Co., Ltd., China), 4% paraformaldehyde (No. 21348860, Beijing Lanjie Technology Co., Ltd., China), trifluoroacetic acid (Shanghai Macklin Biochemical Co., Ltd., China), methanol and acetonitrile (Merck, Germany).

CDCA (No. Z01011LA14), UDCA (No. Y16J7C17898), sodium taurochenodeoxycholate (TCDCA-Na, No. Y09S8K43540), and sodium tauroursodeoxycholate (TUDCA-Na, No. B25J9K64146) were purchased from Shanghai Yuanye Biotechnology Co., Ltd., with a purity of more than 98%. Acetic acid (AA, No. H1808008), propionic acid (PA, No. A1903124), isobutyric acid (IBA, No. K1822187), butyric acid (BA, No. D1917157), isovaleric acid (isovaleric acid, IVA, No. C1919091), and valeric acid (valeric acid, VA, No. G1818016) were purchased from Shanghai Aladdin Biochemical Technology Co., Ltd., with purity greater than 99.5%.

A high-sugar and high-fat feed (formula: basic feed 43.5%, lard 17.5%, casein 13%, sucrose 12%, whole milk powder 10%, calcium hydrogen phosphate 2%, experimental animal premix 2%) was purchased from Jiangsu Synergy Pharmaceutical and Biological Engineering Co., Ltd.

### 2.2 Analysis of the main components of BBP

The contents of CDCA, UDCA, TCDCA, and TUDCA in BBP were determined by HPLC-ELSD (Waters, USA; Alltech, USA). The determination method is provided in Supplementary Method S1.

### 2.3 Animals

SPF male SD rats (body weight 170–200 g) were purchased from Beijing Charles River Experimental Animal Technology Co., Ltd., Beijing, China (SCXK (Jing) 2021-0011). The animals were raised in the barrier environment of the Experimental Animal Center of Nanjing University of Traditional Chinese Medicine, the temperature was (22 ± 2)°C, the relative humidity was (55 ± 10)%, the light was alternated for 12 h, and the rats were free to drink and eat. This experiment was examined and approved by the Animal Ethics Committee of Nanjing University of Traditional Chinese Medicine, and the ethics application number is 202202A046.

After 1 week of adaptive feeding, the rats were randomly divided into a control group (Con, *n* = 10) and the T2DM group (*n* = 48) according to body weight by stratified grouping method. The control group was fed a regular diet, and the T2DM model group was fed a high-sugar and -fat diet. 4 weeks later, the rats fasted for 12 h, and the T2DM model group was intraperitoneally injected with 1% STZ citric acid buffer for 2 consecutive days (0.1 mmol hands, *p*H 4.5°C and 4°C) at a dose of 30 mg/kg, while rats in the control group were intraperitoneally injected with the same amount of sodium citrate buffer. 72 h later (fasting 12 h in advance), fasting blood glucose was measured by tail vein blood sampling. FBG ≥11.1 mmol/L was considered to be successful. 2 rats failed to make the model, and all of them died after continuing to make the model, and a total of 3 rats died after the end of the model. The 45 rats with the successfully established model were randomly divided into 4 groups according to blood glucose by stratified grouping method, which were model group (MOD, *n* = 12), metformin group (MET, 180 mg/kg, *n* = 11), low-dose BBP group (BBP-L, 41.67 mg/kg, *n* = 11), and high-dose BBP group (BBP-H, 83.33 mg/kg, *n* = 11). The rats in the treatment group were given corresponding drugs, while those in the control group and model group were given distilled water by gastric perfusion with a volume of 10 ml/kg once a day for 30 days. Finally, 10 rats survived in the control group, 7 in the model group, 9 in the metformin group, 8 in the low-dose BBP group, and 8 in the high-dose BBP group, with a mortality rate of 32.5%.

### 2.4 Detection of body weight and FBG

The weight of the rats was measured once a week since they were given a high-sugar and high-fat diet. Before administration and 1, 2, 3, and 4 weeks after administration, after fasting for 12 h, fasting blood glucose (FBG) was measured.

### 2.5 Biochemical index detection

The whole blood of rats was kept at room temperature for 2 h and then centrifuged (3,000 rpm for 4 min) to obtain serum. Determination of triglyceride (TG), total cholesterol (T-CHO), low-density lipoprotein cholesterol (LDL-C), high-density lipoprotein cholesterol (HDL-C), aspartate aminotransferase (AST), and alanine aminotransferase (ALT) according to test box instructions (Nanjing Jiancheng Bioengineering Institute, Nanjing, China). Interleukin 6 (IL-6), Interleukin 10 (IL-10), tumor necrosis factor-alpha (TNF-α), and fasting insulin (FINS) were quantified by ELISA kits (Nanjing Lapuda Biotechnology Co., Ltd., Nanjing, China). The insulin resistance index (HOMA-IR) was calculated by a steady-state model.

### 2.6 Histopathological examination

The liver, kidney, spleen, and pancreas were fixed with 4% paraformaldehyde, then dehydrated, embedded, sectioned, and stained with Histopathological Examination. The microscopic images of the sections were observed under the Panorama DESK/MIDI/250/1000 scanner (3DHISTECH, recording).

### 2.7 Metabolomics study on plasma and urine

100 µl plasma samples were mixed with 300 μl cold acetonitrile, while 200 μl urine samples were mixed with 200 μl cold acetonitrile. The mixture was vortexed for the 60s, placed at 4°C for 30 min, and centrifuged at 13,000 rpm at 4°C for 15 min. After the supernatant was concentrated to dryness by vacuum centrifugation, the plasma was dissolved in 165 μl 50% cold acetonitrile and the urine was dissolved in 190 μl 50% cold acetonitrile. After the solution was vortexed and centrifuged again, 2 μl of the supernatant was injected into UPLC-Q-TOF/MS (Waters, USA) for metabolic analysis. Furthermore, equal aliquots of the processed supernatants from each sample as the quality control (QC) sample.

Samples were analyzed by an ACQUITY UPLC BEH C_18_ column (100 mm × 2.1 mm, 1.7 μm, Waters, USA), which was maintained at 35°C. The mobile phase was 0.1% formic acid solution (mobile phase A) and acetonitrile (mobile phase B). The injection volume was 2 μL and the flow rate was 0.4 ml/min. The gradient elution conditions for plasma analysis were as follows: 0.0–3.0 min, 95%–45% A; 3.0–13.0 min, 45%–5% A; 13.0–14.0 min, 5%–95% A; 14.0–15.0 min, 95% A. The gradient elution conditions for urine analysis were as follows: 0.0–8.0 min, 95%–70% A; 8.0–11.0 min, 70%–30% A; 11.0–13.0 min, 30%–5% A; 13.0–14.0 min, 5%–95% A; 14.0–15.0 min, 95% A. The positive and negative ionization modes of electrospray ionization (ESI) mass spectra were adopted, the mass scanning range was 100–1,000 m/z, the capillary voltage was 3.0 kV, the cone voltage was 30 V, the extraction voltage was 2.0 V, the collision energy was 20∼50 eV, the desolvent temperature and ion source temperature were 350°C and 120°C, the desolvent gas flow rate and cone hole gas flow rate were 800 L/h and 50 L/h, the scanning time was 0.3s, and the interval scanning time was 0.02s. Leucine-enkephalin (ESI^+^: 556.2771 *m/z*, ESI^−^: 554.2615 *m/z*) solution was the locked mass solution.

Import the original data into ProgensisQI (Waters, USA) software for denoising, peak alignment, peak picking, standardization, and normalization. The data were analyzed by partial least squares discriminant analysis (PLS-DA) and orthogonal partial least squares discriminant analysis (OPLS-DA) using SIMCA software (version 14.0, Sweden). Variables with *S*-plot ≥.05, *S*-plot ≤−.05 and VIP >1 were selected as potential differential metabolites. *T*-test and non-parametric tests were used to analyze whether there was a significant difference in the relative peak area of biomarkers between the two groups, and the difference metabolites were screened by *p* <.05. Human Metabolic Database (HMDB, http://www.hmdb.ca/) and MetaboAnalyst database (http://www.metaboanalyst.ca/) were used to identify differential metabolism and analyze metabolic pathways. Origin was used to analyze the correlation between biochemical indexes and metabolites by spearman.

### 2.8 IM analysis

According to the E.Z.N.A. ^®^Soil DNA kit (Omega Bio-Tek, Norcross, GA, United States), the total genomic DNA of the microbial community was extracted from the cecal contents of rats. 1% agarose gel electrophoresis was used to detect the quality of extracted genomic DNA, and Nano Drop 2000 (Thermo Scientific Inc., United States) was used to determine the concentration and purity of DNA. The V3-V4 hypervariable region of the bacterial 16s rRNA gene was amplified by PCR (Gene Amp 9700, ABI, United States) with primer 338F (5′-ACT​CCT​ACG​GGA​GGC​AGC​AG-3′) and 806 R (5′-GGACTACHVGGGTWTCTAAT-3′). The PCR amplification procedure was as follows Pre-denatured 3 min at 95°C, 27 cycles (denatured at 95°C for the 30s, annealed at 55°C for 30s, extended at 72°C for 30s), and single extension at 72°C for 10 min. The PCR products were recovered by 2% agarose gel, purified by AxyPrep DNA Gel Extraction Kit (Axygen Biosciences, Union City, CA, United States), quantified by Quantus gel Fluorometer (Promega, United States), and sequenced using Illumina’s MiSeq PE300 platform.

Using UPARSE software (http://drive5.com/uparse/version 7.1), the sequence after quality control splicing was operated by Operational taxonomic unit (OTU) clustering, and chimerism was eliminated according to 97% similarity. The RDP classifier (http://rdp.cme.msu.edu/, version 2.11) ratio was used to annotate the OTU taxonomy of the Silva 16s rRNA gene database with a 70% confidence threshold. Mothur software (http://www.mothur.org/wiki/Calculators) was used to calculate Alpha diversity. Using R language tools to draw principal coordinates analysis (PCA) and principal coordinates analysis (PCoA) diagrams to test the similarity of microbial community structure among samples. Linear discriminant analysis (LDA) was performed on samples of different groups to identify the species that had significant differences in sample classification using linear discriminate analysis effect size (LEfSe) software. The Spearman correlation coefficient between biochemical indexes and IM and between metabolites and IM were evaluated using the R package. The 16s function was predicted and analyzed by using PICRUSt2 (version 2.2.0) software.

### 2.9 Detection of SCFAs in the cecum

The contents of AA, PA, IBA, BA, IVA, and VA were determined by GC-FID (Clarus® 680, PerkinElmer, United States). Detailed experimental steps are provided in Supplementary Method S2.

### 2.10 Statistical analysis

GraphPad Prism 8 software was used for statistical analysis, and One-way ANOVA analysis and the Kruskal-Wallis test were used for comparison between groups. The value of *p* <.05 was statistically significant.

## 3 Results

### 3.1 BBP chemical components analysis

**FIGURE 1:**
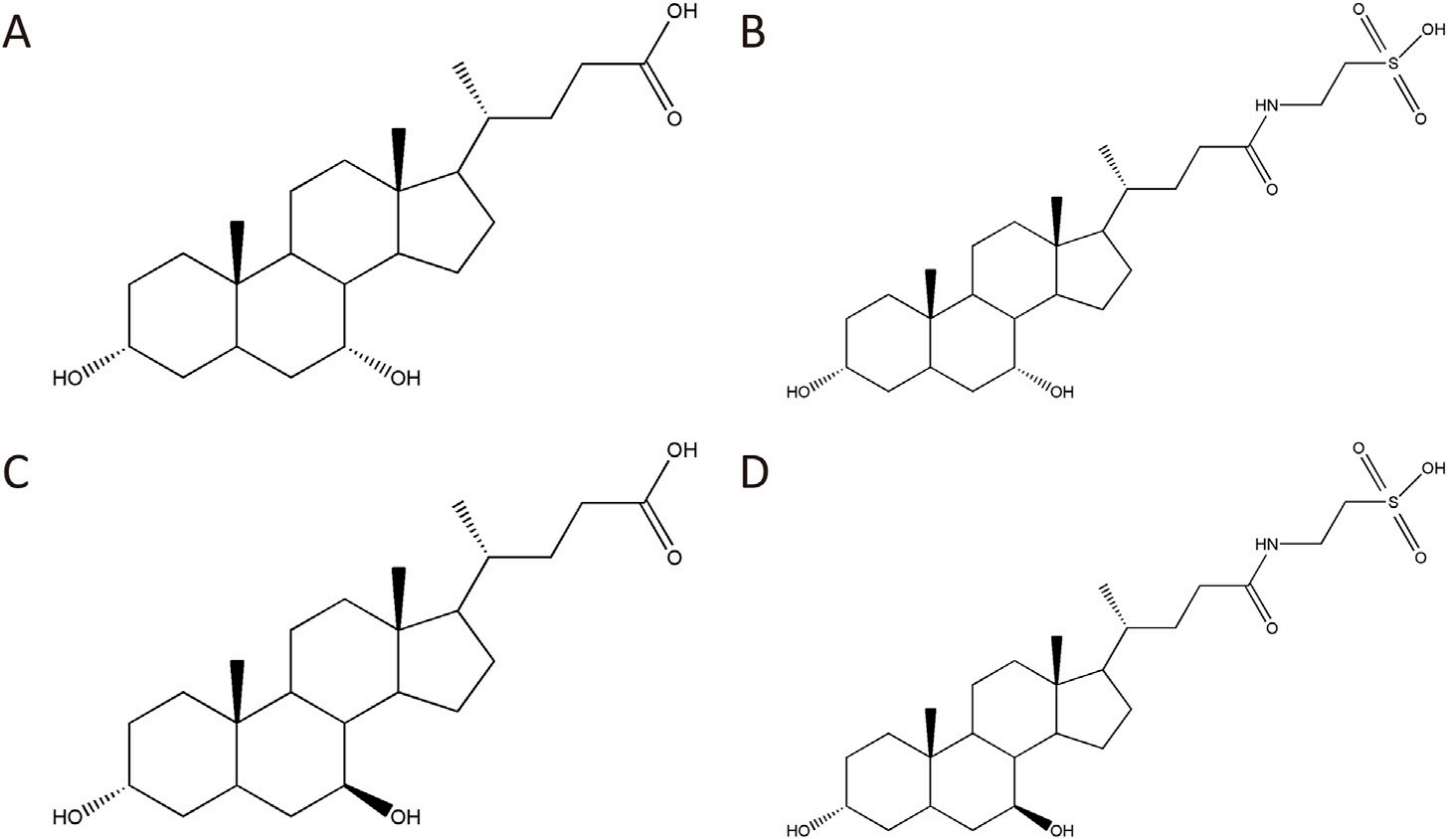
Four main chemical components of BBP: CDCA **(A)**, TCDCA **(B)**, UDCA **(C)**, TUDCA **(D)**.

Four main chemical components of BBP were detected by HPLC-ELSD (Figure 1). The contents of CDCA, UDCA, TCDCA, and TUDCA were 1.95%, 0.59%, 45.78%, and 38.49%, respectively (Supplementary Tables S1, S2; Supplementary Figure S1).

### 3.2 Effects of BBP on body weight and FBG in T2DM rats

**FIGURE 2:**
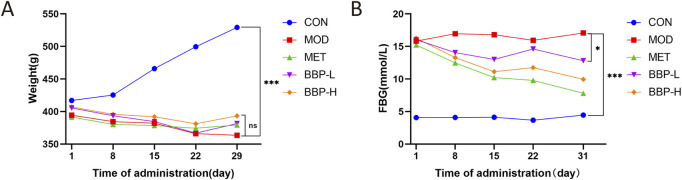
The effects of BBP on body weight **(A)**, FBG **(B)**. Values are presented as the mean ± SD, *n* = 6. *p < .05, *p < .05, **p < .01, ***p < .001: treatment vs. model.

As shown in Figure 2, compared with the control group, the body weight of rats in the model group decreased significantly and the level of FBG increased significantly. Compared with the model group, the body weight of the treatment group had no significant change, and the FBG tended to be adjusted to the control group. It is suggested that BBP can reduce blood glucose in T2DM rats, and the efficacy is enhanced with the increase in dose.

### 3.3 Biochemical index analysis in serum

**FIGURE 3:**
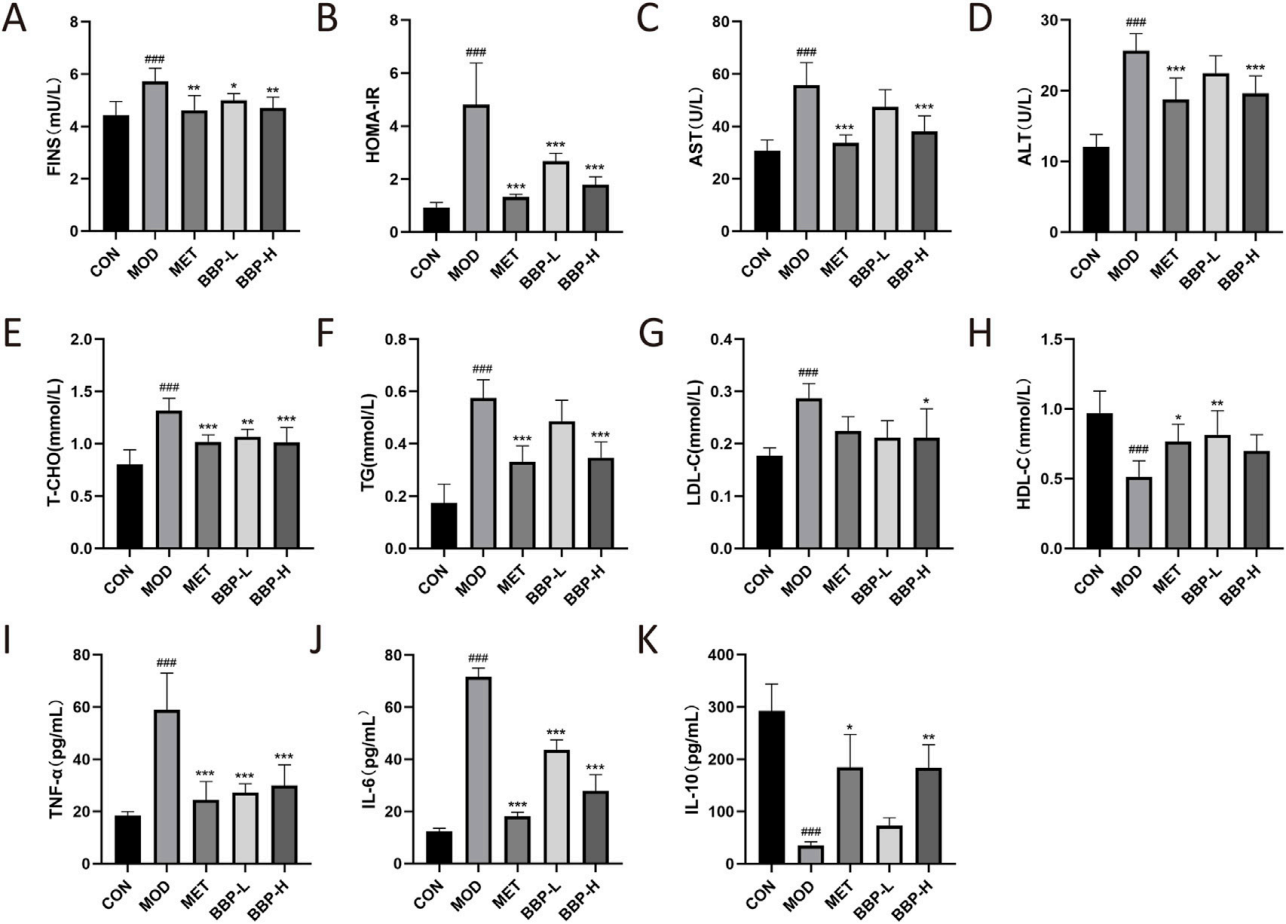
The effects of BBP on FINS **(A)**, HOMA-IR **(B)**, AST **(C)**, ALT **(D)**, T-CHO **(E)**, TG **(F)**, LDL-C **(G)**, HDL-C **(H)**, TNF- *α* **(I)**, IL-6 **(J)**, IL-10 **(K)**. Values are presented as the mean ± SD, *n* = 6. ^#^ *p* < .05, ^##^ *p* < .01, ^###^ *p* < .001: model vs. control; *p < .05, **p < .01, ***p < .001: treatment vs. model.

As shown in Figure 3, compared with the control group, the levels of FINS, HOMA-IR, T-CHO, TG, LDL-C, AST, ALT, IL-6, and TNF-α in the model group were significantly increased, while the levels of HDL-C and IL-10 were significantly decreased. Compared with the model group, the level of FINS and HOMA-IR in each treatment group decreased significantly, indicating that BBP can reduce IR. After administration, the levels of AST and ALT had a downward trend, and the effect of the BBP-H group was better, with a significant difference, suggesting that BBP can improve the liver injury of T2D rats. After administration, the levels of T-CHO, TG, and LDL-C all had a downward trend, among which the effect of the BBP-H group was better, and the difference was significant, while the level of HDL-C was up-regulated, and the effect of the BBP-L group was better, which suggested that T2D rats had the symptom of lipid metabolism disorder, and BBP could improve the blood lipid metabolism of T2D rats. After administration, the levels of IL-6 and TNF-*α* decreased significantly, while the level of IL-10 tended to increase, and the effect of the BBP-H group was better, with a significant difference, indicating that BBP can improve the inflammatory response of T2D rats.

### 3.4 Pathological section analysis

**FIGURE 4:**
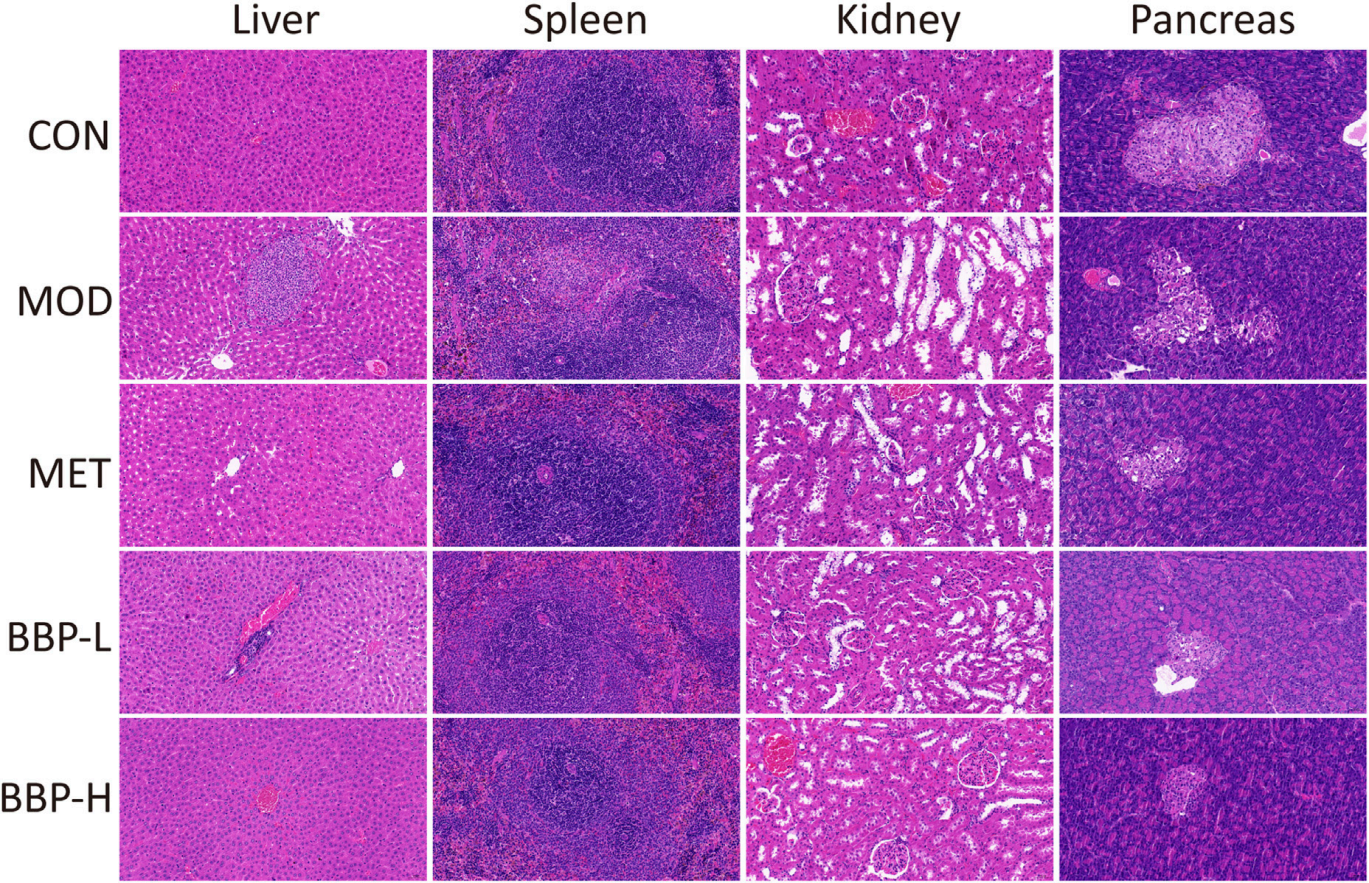
The effects of BBP on histopathology (HE staining × 20) of liver, spleen, kidney and pancreas in rats.

The result of Histopathological Examination staining of the liver, kidney, spleen, and pancreas tissue is shown in Figure 4. Compared with the control group, the arrangement of liver tissue in the diabetic group is more disordered, there are more vascular congestion, hepatocyte necrosis, structure disappearance, and more granulocytes around focal infiltration. After administration, the liver tissue cells of rats were arranged neatly, and the inflammation was improved. Compared with the control group, the spleen tissue of diabetic rats occasionally showed purulent foci, more granulocytes showed focal infiltration, and few necrotic cell fragments and cavity-like structures were seen. After administration, the pathological changes of the spleen in all groups were improved to varying degrees. Compared with the control group, there was more vascular congestion, obvious dilatation of renal tubules, necrosis of renal tubular epithelial cells, deep staining, and fragmentation of nuclei in the diabetic group. After administration, the dilatation of renal tubules and the necrosis of renal tubular epithelial cells were significantly alleviated. Compared with the control group, the number of islets in the diabetic group was less, the size was small, the shape was irregular, few acinar cells infiltrated into the islets, more islet cells were necrotic, and the nucleus pyknosis was deeply stained. After administration, the tissue structure and cell necrosis of rat islets were improved. Therefore, BBP can improve the tissue damage of the liver, spleen, kidney, and pancreas in T2D rats.

### 3.5 Metabolism analysis

**FIGURE 5:**
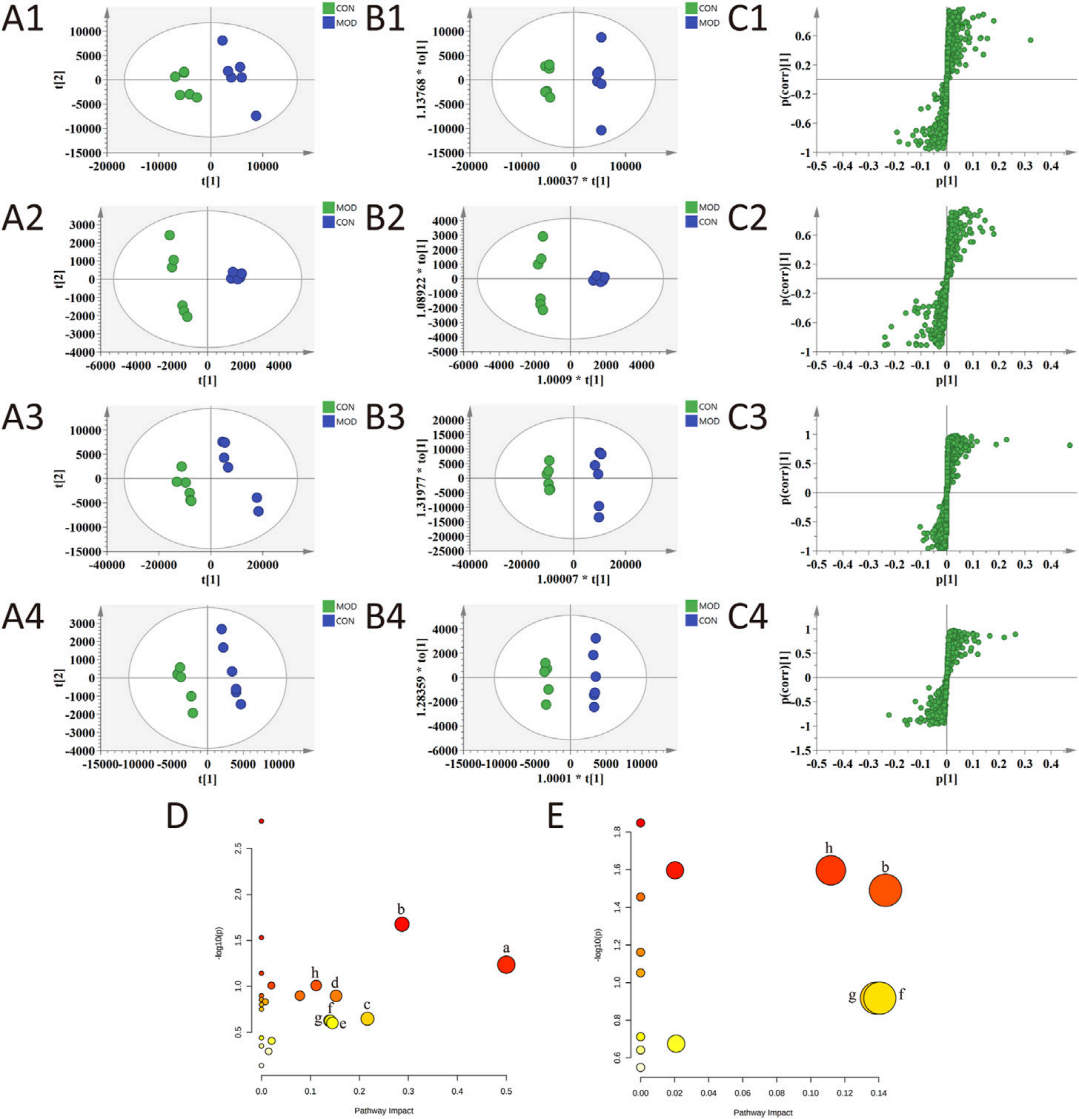
PLS-DA **(A1–A4)**, OPLS-DA **(B1–B4)** and S-plot of OPLS-DA **(C1–C4)** for plasma (1, 2) and urine (3, 4) of model group vs. control group in positive (1, 3) and negative (2, 4) ion mode; metabolic pathways involved in all markers in plasma and urine for T2DM **(D)**; metabolic pathways involved in potential markers in plasma and urine regulated by BBP **(E)**. (a) phenylalanine, tyrosine and tryptophan biosynthesis; (b) tryptophan metabolism; (c) retinol metabolism; (d) tyrosine metabolism; (e) purine metabolism; (f) pentose and glucuronate interconversions; (g) starch and sucrose metabolism; (h) glycerophospholipid metabolism.

**FIGURE 6:**
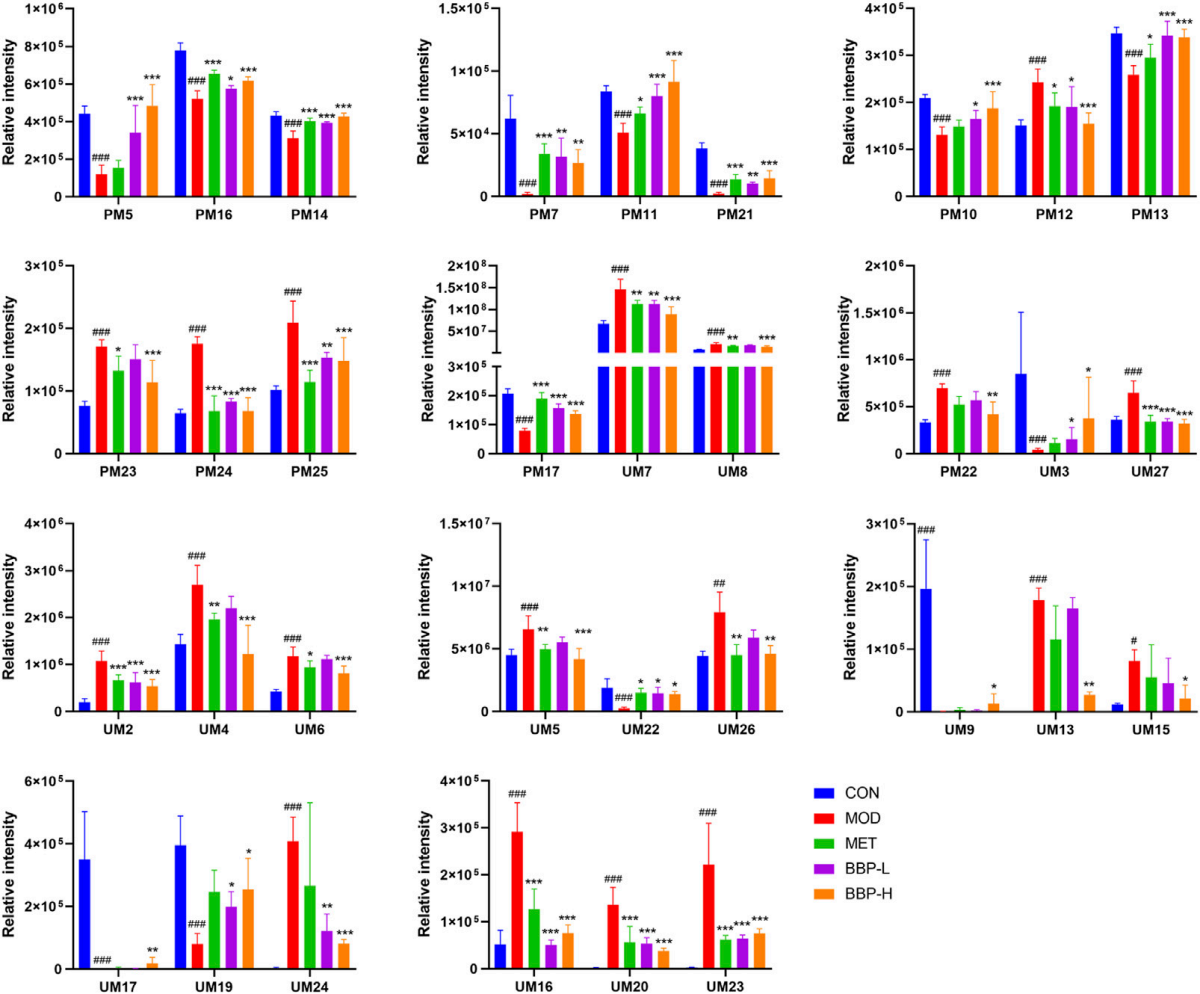
Relative peak areas of potential biomarkers identified in plasma and urine in positive and negative ion modes. Values are presented as the mean ± SD, *n* = 6. ^#^ *p* < .05, ^##^ *p* < .01, ^###^ *p* < .001: model vs. control; *p < .05, **p < .01, ***p < .001: treatment vs. model.

The results of the PLS-DA analysis are shown in Figures 5A1–A4. The plasma and urine samples of the control group and the model group can be separated obviously in both positive and negative ion modes. The results showed that the levels of metabolites in the serum and urine of T2D rats changed significantly, showing metabolic disorders. From the results of the OPLS-DA (Figures 5B1–B4) analysis, *S*-plot (Figures 5C1–C4) can be obtained. The farther the point on *S*-plot is from zero, the greater the contribution to the difference between groups. A total of 55 metabolites were identified by introducing the differential metabolites into the HMDB database, including 28 metabolites from plasma and 27 metabolites from urine. The mass spectrometric data and the changing trend of the model group compared with the control group are shown in Supplementary Table S3. As shown in Figure 6, 33 of these metabolites can be recalled by BBP after administration, which are considered to be potential biometabolites for the hypoglycemic effect of BBP.

The identified differential metabolites were introduced into the MetaboAnalyst database for metabolic pathway analysis (Figures 5D, E). The key metabolic pathways were screened according to the impact value >0.1. The results show that the occurrence of T2DM may be related to many metabolic pathways, including phenylalanine, tyrosine and tryptophan biosynthesis, tryptophan metabolism, retinol metabolism, tyrosine metabolism, purine metabolism, pentose and glucuronate interconversions, starch and sucrose metabolism, and glycerophospholipid metabolism. The metabolic pathways of the hypoglycemic effect of BBP are tryptophan metabolism, pentose and glucuronate interconversions, starch and sucrose metabolism, and glycerophospholipid metabolism. It is suggested that BBP administration can improve the metabolic disorder of T2DM by changing the metabolic pathway and related biomarkers.

### 3.6 IM analysis

#### 3.6.1 Diversity analysis

**FIGURE 7:**
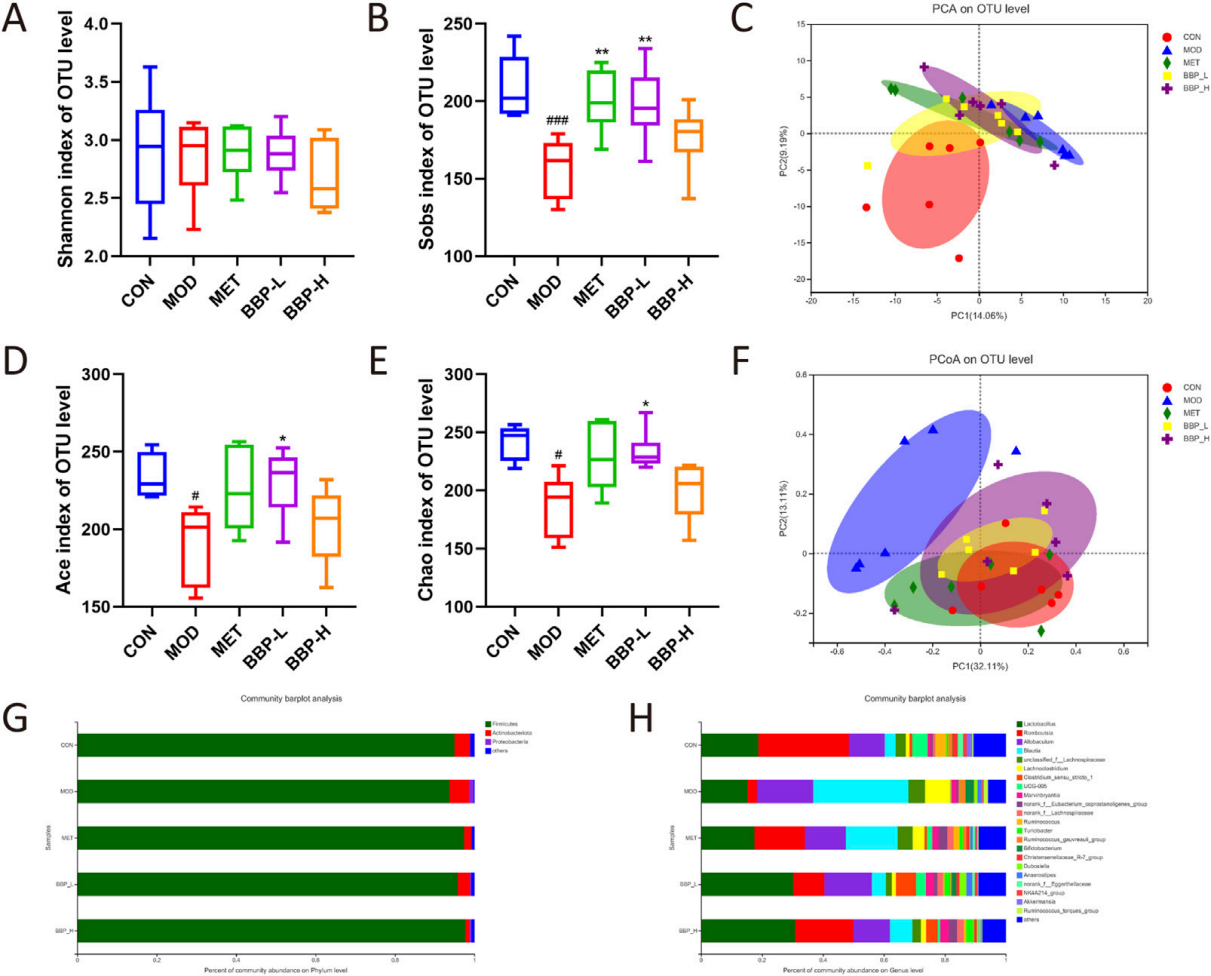
The results of microbial diversity and community composition analysis. Shannon index **(A)**, Sobs index **(B)**, PCA analysis **(C)**, Ace index **(D)**, Chao indexes **(E)**, PCoA analysis **(F)**. The percent of community abundance at the phylum **(G)** and genus **(H)** levels. Values are presented as the mean ± SD, *n* = 6. ^#^ *p* < .05, ^##^ *p* < .01, ^###^ *p* < .001: model vs. control; *p < .05, **p < .01, ***p < .001: treatment vs. model.

As shown in Figures 7A, B, D, E, there was no significant difference in the Shannon index representing diversity in the cecal content samples, but the Sobs, Ace, and Chao indexes representing the richness of IM in the control group were significantly higher than those in the model group, which was restored after treatment, indicating that BBP could improve the richness of IM in T2DM rats. PCA and PCoA diagrams showed that the control group was separated from the model group (Figures 7C, F). The administration group deviated from the model group to some extent and approached the control group, indicating that BBP caused changes in the community composition of IM.

#### 3.6.2 Community composition analysis

The analysis of the composition of rat IM at the phylum and genus level is shown in Figures 7G, H. At the phylum level, the main classifications in each group of samples are *Firmicutes*, *Actinobacteria*, and *Proteobacteria*. Compared with the control group, the abundance of *Firmicutes* in the model group decreased, while the abundance of *Actinobacteria* and *Proteobacteria* increased. At the genus level, the main classifications are *Lactobacillus*, *Romboutsia*, *Allobaculum*, and *Blautia*. Compared with the control group, the proportion of *Lactobacillus*, and *Romboutsia* decreased and the proportion of *Allobaculum* and *Blautia* increased in the model group. After administration, the composition of IM in rats continued to approach the control group, indicating that BBP can improve the disorder of IM in T2DM rats to some extent.

#### 3.6.3 Screening of differential bacteria and regulation of BBP

**FIGURE 8:**
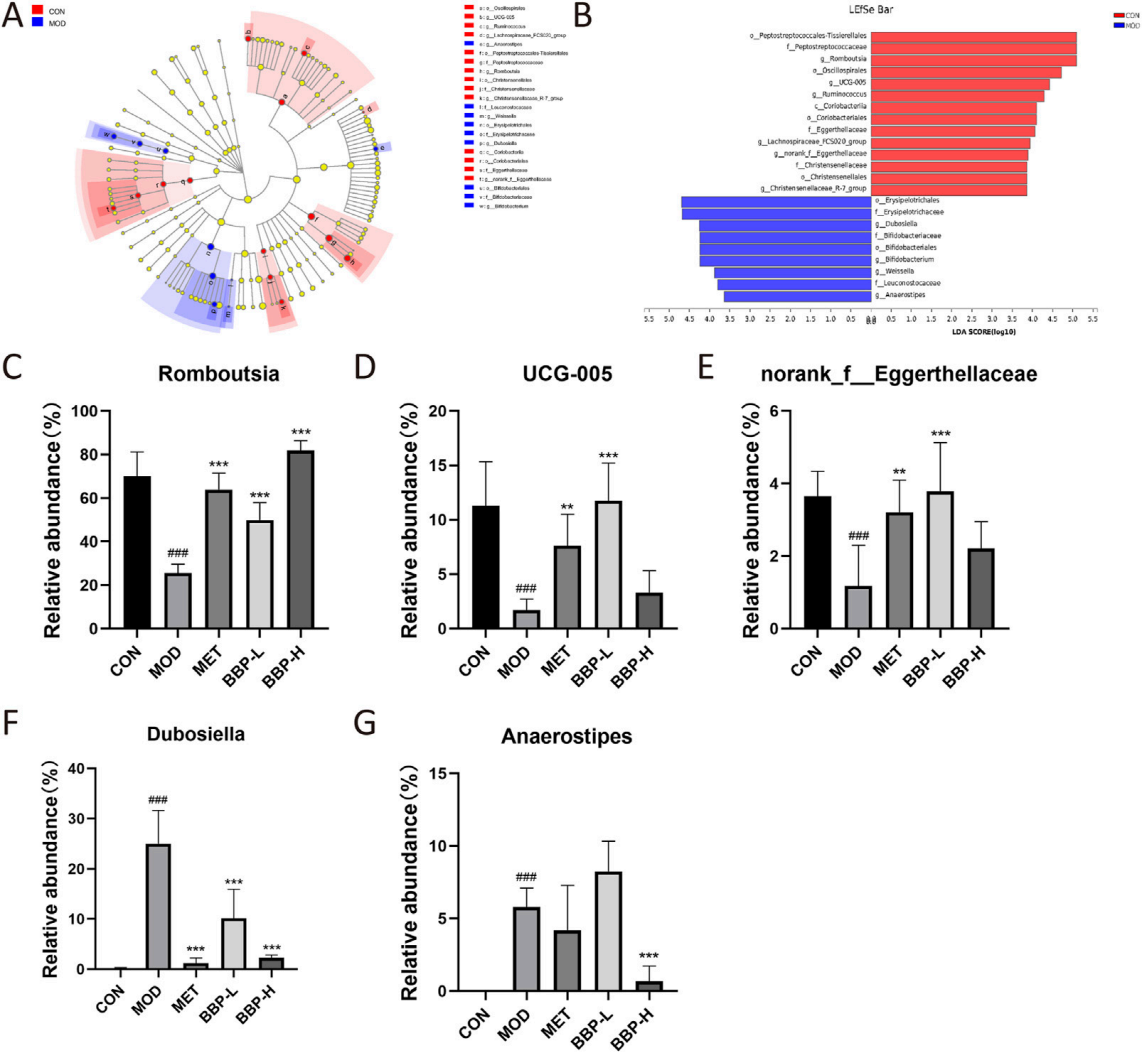
The results of screening differential bacteria and regulating effect of drugs. LEfSe dendrogram **(A)**, LDA discriminant bar graph **(B)**. Relative abundance of five flora at genus level in each group**(C–G).** Values are presented as the mean ± SD, *n* = 6. ^#^ *p* < .05, ^##^ *p* < .01, ^###^ *p* < .001: model vs. control; *p < .05, **p < .01, ***p < .001: treatment vs. model.

The differential flora obtained by LEfSe analysis is shown in Figure 8. A total of 10 species with significant differences at the genus level were identified and statistically analyzed to compare the relative abundance of IM in each group after administration. The results showed that compared with the control group, the abundance in the model group increased significantly in *Dubosiella* and *Anaerostipes*, and decreased significantly in *Romboutsia*, *UCG-005*, and *norank_f__Eggerthellaceae*. Each administration group had different degrees of callback effect.

#### 3.6.4 Functional prediction of IM

**FIGURE 9:**
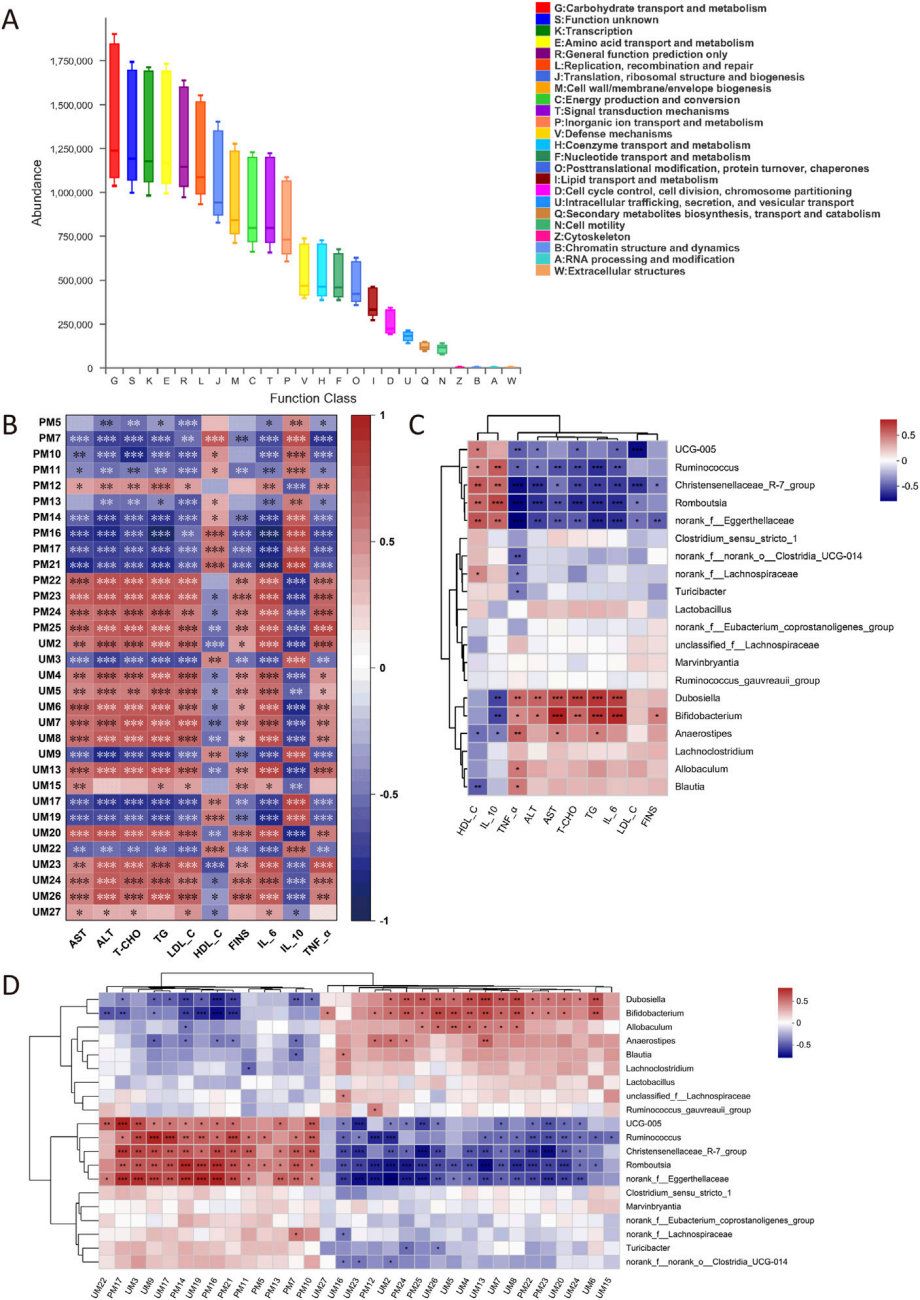
COG function prediction **(A)**. Correlation analysis between biochemical indexes and metabolites **(B)**. Correlation analysis between biochemical indexes and IM **(C)**. Correlation analysis between metabolites and IM **(D)**. The correlation *p < .05, **p < .01, ***p < .001, *n* = 6.

The prediction of COG function is shown in Figure 9A, in which carbohydrate transport and metabolism, transcription and amino acid transport, and metabolic pathways are dominant in the sample.

### 3.7 Relativity analysis

The correlation analysis between biochemical indexes and metabolites is shown in Figure 9B. LysoPC (22:6 (4Z, 7Z, 10Z, 13Z, 16Z, 19Z)/0:0), 5-HT, and NAS were positively correlated with FINS, dyslipidemia, inflammatory factors, and liver injury. PC (22:5(7Z, 10Z, 13Z, 16Z, 19Z)/14:0) and other Lysophosphatidylcholines (lysoPCs) were negatively correlated with these biochemical indexes. The correlation analysis between biochemical indexes and IM is shown in Figure 9C. *UCG-005*, *Ruminococcus*, *Christensenellaceae_R-7_group*, *Romboutsia*, and *norank_f__Eggerthellaceae* were negatively correlated with FINS, dyslipidemia, inflammation, and liver injury. *Dubosiella*, *Bifidobacterium*, and *Anaerostipes* were positively correlated with these biochemical indexes. The correlation analysis between metabolites and IM is shown in Figure 9D. *UCG-005*, *Ruminococcus*, *Christensenellaceae_R-7_group*, *Romboutsia*, and *norank_f__Eggerthellaceae* were negatively correlated with LysoPC (22:6 (4Z, 7Z, 10Z, 13Z, 16Z, 19Z)/0:0), 5-HT, and NAS, and positively correlated with PC and other lysoPCs. *Dubosiella*, *Bifidobacterium*, and *Anaerostipes* were positively correlated with LysoPC (22:6 (4Z, 7Z, 10Z, 13Z, 16Z, 19Z)/0:0), 5-HT and NAS, and negatively correlated with PC and the rest of lysoPCs.

### 3.8 Effect of BBP on SCFAs in cecal contents of T2DM rats

**FIGURE 10:**
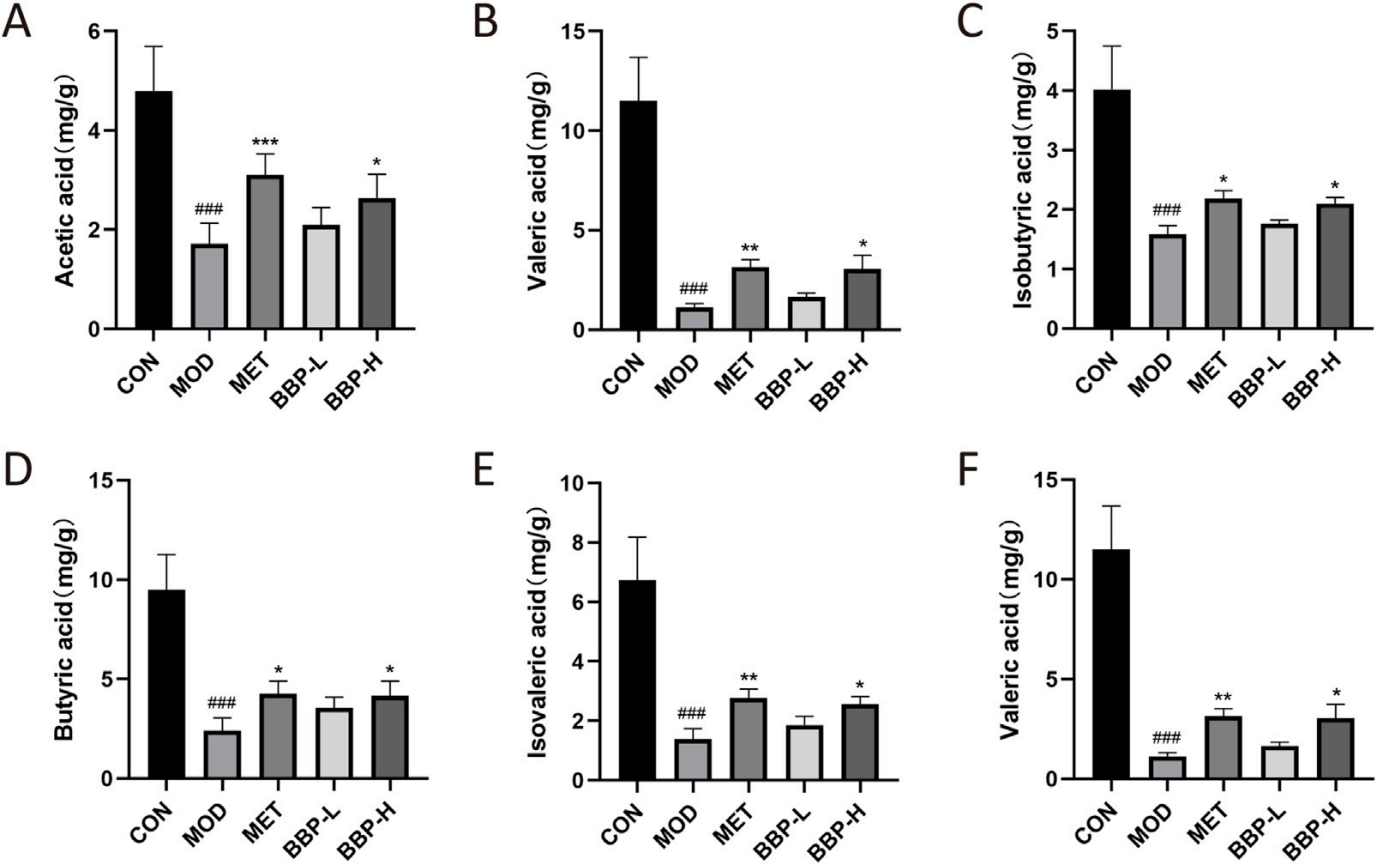
Contents of SCFAs in cecum contents of rats in each group. Acetic acid **(A)**, Propionic acid **(B)**, Isobutyric acid **(C)**, Butyric acid **(D)**, Isovaleric acid **(E)**, Valeric acid **(F)**. Values are presented as the mean ± SD, *n* = 6. ^#^ *p* < .05, ^##^ *p* < .01, ^###^ *p* < .001: model vs. control; *p < .05, **p < .01, ***p < .001: treatment vs. model.

The contents of six SCFAs in the cecum of T2DM rats were determined by GC-FID (Supplementary Tables S4, S5; Supplementary Figure S2). The contents of six SCFAs in cecal contents are shown in Figure 10. Compared with the control group, the level of AA, PA, IBA, BA, IVA, and VA in the model group decreased significantly. After administration, the above concentrations tended to increase, and their concentrations increased with the increase in drug dose. It is suggested that BBP can restore the decrease of SCFAs in the feces of T2DM rats.

## 4 Discussion

The clinical features of 2TDM include elevated glucose level, IR, a disorder of fat metabolism, organ injury, and inflammatory reaction. In this study, the hypoglycemic effect of BBP was evaluated by detecting FBG, biochemical indexes, histopathology, and calculating HOMA-IR in rats. Insulin in T2DM reduces glucose uptake and utilization, and high blood glucose levels in the blood will cause islet cells to release excess insulin, resulting in IR. In this study, BBP can reduce the FBG, FINS, and HOMA-IR index of T2DM rats, and significantly improve the morphology of islets, indicating that BBP can alleviate IR and improve the stability of blood glucose. T2DM is often accompanied by lipid metabolism disorder, and IR is the central link leading to dyslipidemia. In the presence of IR, a large number of lipids are decomposed, resulting in high levels of TC, TG, and LDL-C in circulation and a decrease in HDL-C levels (Yang et al., 2018; Jin et al., 2022). In this study, BBP can significantly reduce the levels of serum TC, TG, and LDL-C and increase the level of HDL-C, indicating that BBP can reduce the lipid accumulation of T2DM mice. Serum ALT and AST are important markers of liver injury, and the increase in their activity will lead to liver fat accumulation and IR, which can induce the occurrence and development of diabetes (Liu et al., 2017; Shi et al., 2022). In this study, BBP significantly decreased the activities of serum ALT and AST, and the liver tissue injury was significantly improved, indicating that BBP can alleviate the liver injury caused by T2DM. Inflammatory response participates in the whole process of the development of 2TDM. A large number of inflammatory mediators such as TNF-α and IL-6 are produced and released, which interfere with insulin signal transduction and lead to IR (Zhang et al., 2018). IL-10 is an important anti-inflammatory cytokine, which can inhibit the release of TNF-α and IL-6 by cells, which is particularly important in maintaining intestinal microbial immune homeostasis (Neumann et al., 2019). In this study, BBP increased the activity of IL-10, decreased the content of IL-6 and TNF-α, and improved the focal infiltration of organs, indicating that BBP has an anti-inflammatory effect.

5-hydroxytryptamine (5-HT, serotonin) and N-Acetylserotonin (NAS) are the intermediates of tryptophan metabolism. More than 90% of 5-HT is a gastrointestinal hormone synthesized and released in enterochromaffin cells, which plays a key role in glucose homeostasis (Mao et al., 2019). IR during pregnancy can significantly increase the expression of 5-HT. 5-HT thus promotes insulin secretion in *β*-cells through the 5-HT3 receptor, and this effect becomes more severe under metabolic stress induced by a high-fat diet (Kim et al., 2015). NAS is transformed from 5-HT and has the effects of anti-inflammation and antioxidation (Nishida et al., 2002; Kang et al., 2022). In this study, the contents of 5-HT and NAS in the model group were up-regulated and down-regulated in varying degrees after the administration of BBP. It is suggested that the regulation of tryptophan metabolism is the potential mechanism of BBP in the treatment of diabetes, and the increase of NAS content may be the same as 5-HT, due to metabolic compensation due to homeostasis disorder. LysoPC and phosphatidylcholine (PC) are the intermediates of glycerophosphate metabolism. PC is synthesized in the endoplasmic reticulum, which provides the membrane needed for protein synthesis and output, and participates in lipid storage/secretion, oxidative stress, and diabetes (Lagace and Ridgway, 2013). LysoPCs are hydrolyzed by PC under the action of phospholipase and participate in a variety of biological functions such as vascular endothelial dilation, cellular immune response, and insulin secretion (Yamamoto et al., 2021). It can be used as a circulatory metabolic intermediate related to inflammation and oxidative stress to predict the risk of early diabetes (Ha et al., 2012). In this study, except LysoPC (22:6 (4Z, 7Z, 10Z, 13Z, 16Z, 19Z)/0:0), the contents of lysoPCs and PC in the model group were all down-regulated, which revealed the metabolic disorder of glycerophosphate in T2DM rats. After the administration of BBP, the contents of differential metabolites were all adjusted to different degrees. These results suggest that the potential mechanism of BBP in the treatment of diabetes is the regulation of glycerol phospholipid metabolic dysfunction.

There is a complex and close relationship between IM and bile acid. IM regulates the synthesis, metabolism, and reabsorption of bile acid, while the composition of the bile acid pool regulates the diversity and dynamic balance of IM. The imbalance between them is involved in the occurrence and development of T2DM (Guo et al., 2022). Bile acid can exert a direct antibacterial effect on IM by destroying bacterial cell membranes and indirectly producing antimicrobial peptides mediated by the farnesoid X receptor (Ridlon et al., 2014). Therefore, the normal level of bile acid can stimulate the growth of bacteria through bile acid metabolic enzymes, inhibit the excessive growth of bile acid-sensitive bacteria and potential pathogens, and prevent bacterial migration, thus maintaining the dynamic balance of IM and intestinal barrier function (Guo et al., 2022).

*Lactobacillus* is a microorganism living in the body that is beneficial to the health of the host. It can improve the disorder of lipid metabolism and IR in patients with diabetes, and further assist in the control of blood glucose levels (Tiderencel et al., 2020; Rodrigues et al., 2021). *Lactobacillus* has also been found to reduce intestinal leakage and inflammation (Wang et al., 2020). *Allobaculum* can produce trimethylamine oxide (Zhu et al., 2016), while trimethylamine oxide cann't only promote fat production by inhibiting BA-mediated hepatic farnesoid X receptor signal (Tan et al., 2019), but also promote IR, thereby affecting blood glucose homeostasis and the occurrence and development of T2DM (Chen et al., 2019). In this experiment, the abundance of *Lactobacillus* was down-regulated and *Allobaculum* was up-regulated in the model group, which was consistent with the results of the study. Studies have shown that reducing the abundance of *Blautia* may help to reduce obesity, reduce blood lipid levels and regulate blood glucose homeostasis (Zhao et al., 2022). *Dubosiella* was positively correlated with liver injury, lipid metabolism, and fibrosis (Zhuge et al., 2022). Other studies have shown that the intestinal disorder induced by diabetes can be reversed by increasing the abundance of *Blautia* and *Dubosiella* (Pan et al., 2022). *Dubosiella* can also regulate the production of SCFAs, thus alleviating the development of obesity (Qiu et al., 2021). Intestinal *Anaerostipes* have a potentially beneficial effect and can reduce fasting blood glucose levels (Bui et al., 2021). However, other research data show that *Anaerostipes* were abundant in diabetic nephropathy and diabetes (Du et al., 2021). In this experiment, *Blautia*, *Dubosiella*, and *Anaerostipes* in the model group all showed an upward trend. The abundance of bacteria in T2DM rats may not be constant, and the compensatory growth of bacteria has dynamic changes, which need to be verified by further study.

SCFAs are the main bacterial metabolite produced by IM fermenting dietary fiber, including AA, PA, BA, and VA (Tan et al., 2014). Branched-chain fatty acids IBA and IVA are produced in the catabolism of branched-chain amino acids (Macfarlane and Macfarlane, 2003). SCFAs act as an energy substrate in the tricarboxylic acid cycle, regulating hormones related to satiety regulation and insulin secretion, as well as regulating the physiological status of immune cells and microglia to promote inflammatory regulation, energy balance and glucose balance (Luo et al., 2022). Studies have shown that butyrate can reduce energy intake and promote fat oxidation by activating brown adipose tissue (Li et al., 2018). Butyrate may also activate intestinal gluconeogenesis through different gene expression and hormone regulatory pathways, thus improving insulin sensitivity (Hartstra et al., 2015). PA is mainly absorbed by the liver and is a good precursor of gluconeogenesis, adipogenesis, and protein synthesis, while acetate enters the peripheral circulation and is metabolized by peripheral tissue, which is the substrate of cholesterol synthesis (Schwiertz et al., 2009). *UCG-005* may regulate hormone levels and inflammatory expression in the pancreas by increasing the production of AA and BA, and further improving islet *β*-cell dysfunction, thereby alleviating hyperglycemia and IR (Li et al., 2021). *Romboutsia* plays a key role in maintaining host health. *UCG-005* interacts with it to promote the production of SCFAs (Li J et al., 2022). A large number of *Romboutsia* and *Lactobacillus* also play a positive role in the production and immune regulation of SCFAs (Li et al., 2019; Wu et al., 2020). Studies have shown that when glucose and lipid metabolism disorders occur, the homeostasis of related IM will be destroyed, thus reducing the production of SCFAs (Canfora et al., 2015). In this study, the contents of SCFAs in the model group were significantly lower than those in the blank group, and BBP supplementation significantly increased the content of SCFAs, especially the contents of BA and VA, which may be caused by increasing the abundance of SCFAs-producing bacteria (*Lactobacillus*, *Romboutsia*, *UCG-005*). It can be seen that SCFAs may help to alleviate the inflammatory reaction, lipid metabolism disorder, and IR caused by T2DM.

Metabonomic studies have shown that the metabolic pathways related to T2DM are mainly related to amino acid metabolism and carbohydrate transport and metabolism, which is consistent with the prediction of IM function. Previous analysis shows that the relationship between biochemical indexes, metabolites, and IM are closely related, which is of great significance to the occurrence and treatment of T2DM. *Dubosiella* was positively correlated with liver injury and lipid metabolism (Zhuge et al., 2022). *Romboutsia* and *UCG-005* were negatively correlated with inflammation (Li et al., 2021; Wang et al., 2022). *Christensenellaceae_R-7_group* can relieve IR (Chen et al., 2021). Five-HT can promote insulin secretion (Kim et al., 2015). The relevant analysis results in this study are consistent with the previous analysis results, these findings further suggest that BBP treatment can improve T2DM related indicators by regulating IM components and metabolites.

**FIGURE 11:**
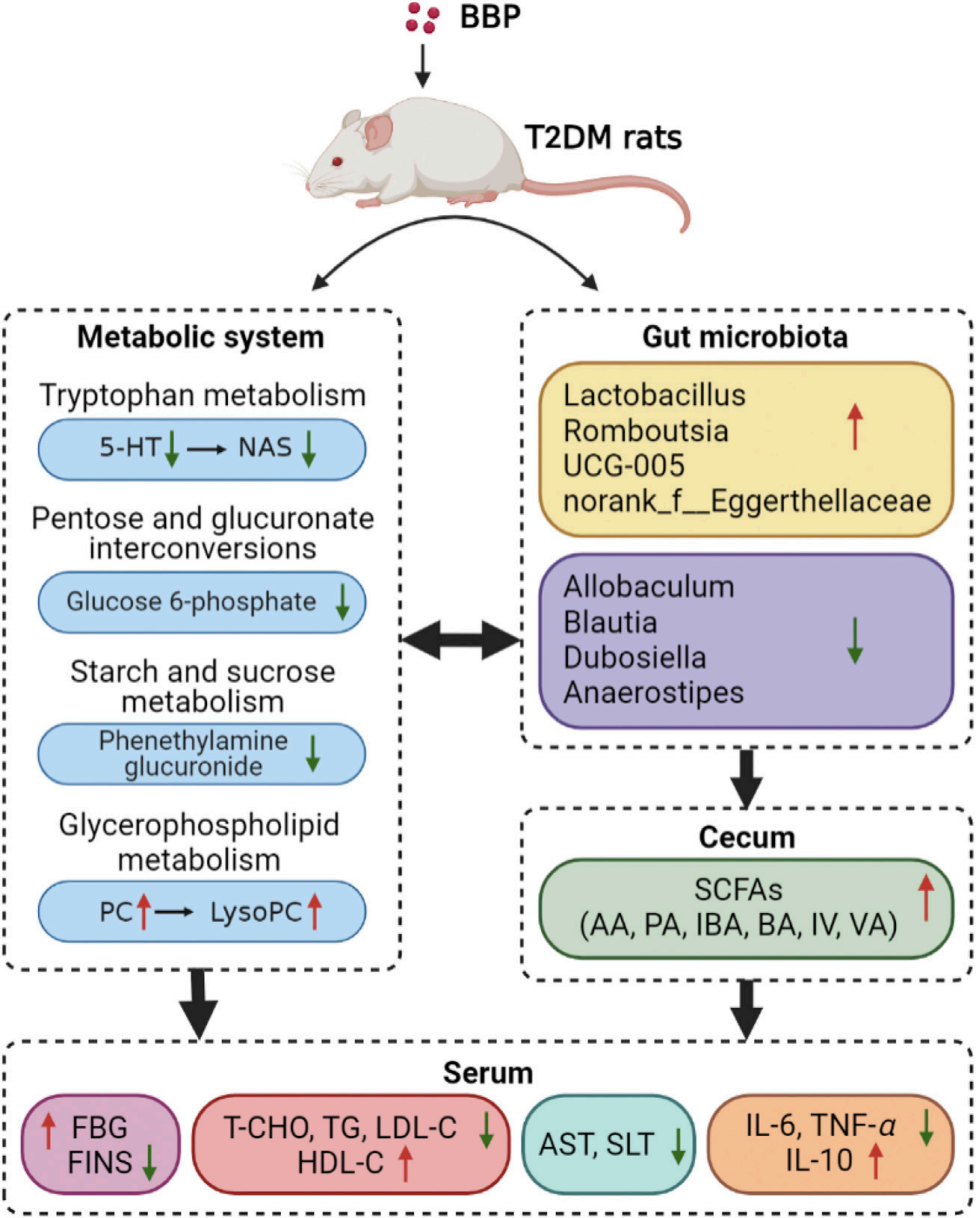
Mechanism of BBP in the treatment of T2DM rats by regulating metabolic pathway, IM composition and SCFAs level (figure was created with BioRender.com).

To sum up, our results suggest that BBP can regulate blood glucose homeostasis in T2DM rats induced by a high-sugar and high-fat diet combined with STZ. As shown in Figure 11, the protective effect of BBP may be achieved by inhibiting inflammatory response, protecting against organ damage, reducing IR, and lipid metabolic disorders. In addition, BBP slows down the development of diabetes by regulating metabolic disorders, restoring the composition of IM, and increasing the content of SCFAs. These findings provide new insights into the potential hypoglycemic mechanism of BBP and contribute to the development and utilization of BBP resources.

## Data Availability

The original contributions presented in the study are publicly available. This data can be found here: https://www.ncbi.nlm.nih.gov/sra. Accession number: PRJNA898677.

## Glossary

5-HT: 5-hydroxytryptamine
AA: acetic acid
ALT: alanine aminotransferase
AST: aspartate aminotransferase
BA: butyric acid
BBP: bear bile powder
CDCA: chenodeoxycholic acid
FBG: fasting blood glucose
FINS: fasting insulin
HDL-C: high-density lipoprotein cholesterol
HOMA-IR: insulin resistance index
IBA: isobutyric acid
IL-10: interleukin 10
IL-6: interleukin 6
IM: intestinal microbiota
IR: insulin resistance
IVA: isovaleric acid
LDA: linear discriminant analysis
LDL-C: low-density lipoprotein cholesterol
LEfSe: linear discriminate analysis effect size
lysoPCs: lysophosphatidylcholines
NAS: N-Acetylserotonin
OPLS-DA: orthogonal partial least squares discriminant analysis
OUT: operational taxonomic unit
PA: propionic acid
PC: phosphatidylcholine
PCA: principal coordinates analysis
PCoA: principal co-ordinates analysis
PLS-DA: partial least squares discriminant analysis
SCFAs: short-chain fatty acids
STZ: streptozotocin
T2DM: type 2 diabetes mellitus
TCDCA: taurochenodeoxycholic acid
T-CHO: total cholesterol
TG: triglyceride
TNF-α: tumor necrosis factor alpha
TUDCA: tauroursodeoxycholic acid
UDCA: ursodeoxycholic acid
VA: valeric acid
