# Supplementary material for: Bear bile powder ameliorates type 2 diabetes via modulation of metabolic profiles, gut microbiota, and metabolites
Source: Front Pharmacol. 2023 Jan 4;13:1090955. doi: 10.3389/fphar.2022.1090955 (PMC9846258; doi:10.3389/fphar.2022.1090955)
Supplement: Supplementary file 1 [file DataSheet1.PDF]

Supplemental material

1 Supplementary Data

Method S1 Analysis of the main components of BBP

The quantitative analysis of the composition of BBP extract was performed on HPLC-ELSD (Waters, USA; Alltech, USA). The chromatographic column was Agilent ZORBAX SB C18 (250mm × 4.6 mm,5 μm , Waters, USA). The mobile phase was 0.05% Trifluoroacetic acid (mobile phase A) and acetonitrile (mobile phase B). Gradient elution as follows: 0 ~ 4 min (90% ~ 60% A), 4 ~ 22 min (60% ~ 40% A), 22 ~ 23 min (40% ~ 90% A), 23 ~ 29 min (90% A). Flow rate: 1mL / min; column temperature: 30 ℃; injection volume: 10 μL; ELSD: drift tube 80 ℃; nitrogen flow rate: 2.5L / min.

Method S2 Short-chain fatty acid analysis of cecum contents

The short-chain fatty acid content determination of cecum contents was performed on GC-FID (Clarus® 680, PerkinElmer, USA). In detail, chromatographic separations were performed on a HP-5MS capillary column (30 m × 0.250 mm, 0.25 μ m, Agilent Technologies, Inc., USA). The injection volume was 2μL, and the conditions were as follows: channel sampling rate, 12.5 min/s; carrier gas, helium; split ratio, 10:1; air flow rate, 400 ml/min; H2 flow rate,40 ml/min. The injector temperature was 250 °C, FID detector temperature was 250 °C, and the oven ramp was programmed: the initial temperature was 100 °C, holding for 2.5 min, then heating to 230 °C at a rate of 50 °C/min and holding for 2 minutes.

2 Supplementary Figures and Tables

Table S1 Investigation of the BBP linear relationship

Table 1 Investigation of linear relationship

Compound	Linear equation	R2	Linear range（μg/ml）
CDCA	y=1.5141x+2.5943	0.9971	63~630
UDCA	y=1.5436x+2.4124	0.9978	59~590
TCDCA	y=1.4277x+2.9294	0.9953	67~670
TUDCA	y=1.3468x+3.2103	0.9962	64~640

Table S2 Precision, repeatability, stability and recovery (n=6) of BBP

Compound	Precision (RSD/%)	Repeatability (RSD/%)	Stability (RSD/%)	Recovery（%）	
				Mean	RSD
CDCA	2.07	3.09	4.24	99.27	2.77
UDCA	2.40	2.94	3.84	94.26	2.85
TCDCA	2.59	2.76	3.91	103.33	2.88
TUDCA	1.68	2.99	3.36	97.06	0.99

Table S3 Potential biomarkers selected and identified between model and control rats

No. a	tR (min)	Mass (m/z)	Metabolite	Trend b	Ion mode	HMDB
PM1	6.06	283.1447	Leucylleucine	↑	+	HMDB0028933
PM2	6.93	365.1115	Isomaltose	↑	+	HMDB0002923
PM3	9.45	551.3132	Deoxycholic acid 3-glucuronide	↓	+	HMDB0002596
PM4	9.75	257.1033	5-Methoxydimethyl tryptamine	↓	+	HMDB0002004
PM5	14.41	780.5550	PC (22:5(7Z,10Z,13Z,16Z,19Z)/14:0)	↓	+	HMDB0008689
PM6	6.14	464.3026	Glycocholic acid	↑	-	HMDB0000138
PM7	7.25	391.2868	Deoxycholic acid	↓	-	HMDB0000626
PM8	8.67	512.3003	LysoPC (14:0/0:0)	↓	-	HMDB0010379
PM9	9.01	538.3152	LysoPC (16:1(9Z)/0:0)	↓	-	HMDB0010383
PM10	9.24	588.3306	LysoPC (20:4(8Z,11Z,14Z,17Z)/0:0)	↓	-	HMDB0010396
PM11	9.24	528.3104	LysoPC (20:4(5Z,8Z,11Z,14Z)/0:0)	↓	-	HMDB0010395

PM12	9.37	612.3301	LysoPC (22:6(4Z,7Z,10Z,13Z,16Z,19Z)/0:0)	↑	-	HMDB0010404
PM13	9.42	528.3096	Lithocholyltaurine	↓	-	HMDB0000722
PM14	9.74	480.3093	LysoPC (15:0/0:0)	↓	-	HMDB0010381
PM15	10.01	255.2322	Palmitic acid	↑	-	HMDB0000220
PM16	10.02	540.3303	LysoPC (16:0/0:0)	↓	-	HMDB0010382
PM17	10.72	554.3465	LysoPC (17:0/0:0)	↓	-	HMDB0012108
PM18	10.87	299.2009	12-KETE	↑	-	HMDB0013633
PM19	11.61	594.3786	LysoPC (20:1(11Z)/0:0)	↓	-	HMDB0010391
PM20	11.66	301.2172	Eicosapentaenoic acid	↓	-	HMDB0001999
PM21	11.91	522.3584	LysoPC (18:0/0:0)	↓	-	HMDB0010384
PM22	12.15	327.2327	Docosahexaenoic acid	↑	-	HMDB0002183
PM23	12.15	283.2431	DG (16:0/16:0/0:0)	↑	-	HMDB0007098
PM24	12.53	329.2485	Docosapentaenoic acid (22n-3)	↑	-	HMDB0006528
PM25	13.19	331.26	Adrenic acid	↑	-	HMDB0002226

		42				
PM26	13.45	281.2481	Oleic acid	↑	-	HMDB0000207
PM27	13.45	381.1743	Pyridoxamine	↑	-	HMDB0001431
PM28	14.93	885.5528	PI (18:0/20:4(5Z,8Z,11Z,14Z))	↑	-	HMDB0009815
UM1	0.74	153.0431	Xanthine	↓	+	HMDB0000292
UM2	4.4	369.051	5-Hydroxyisourate	↑	+	HMDB0030097
UM3	9.7	238.0891	Biopterin	↓	+	HMDB0000468
UM4	10.05	261.0441	Glucose 6-phosphate	↑	+	HMDB0001401
UM5	10.51	365.1117	Melibiose	↑	+	HMDB0000048
UM6	13.12	363.1122	Galactosylhydroxylysine	↑	+	HMDB0000600
UM7	13.14	437.2092	N-Acetylserotonin	↑	+	HMDB0001238
UM8	13.14	298.133	Phenethylamine glucuronide	↑	+	HMDB0010323
UM9	1.85	278.0704	5'-Methylthioadenosine	↓	-	HMDB0001173
UM10	3.3	222.0805	N-Acetyl-L-tyrosine	↓	-	HMDB0000866

UM11	3.64	162.0555	L-Tyrosine	↓	-	HMDB0000158
UM12	3.78	393.0532	Inosinic acid	↑	-	HMDB0000175
UM13	4.46	113.0233	2-Acetolactate	↑	-	HMDB0006833
UM14	4.82	385.1162	3,4-Dihydroxyphenylglycol	↓	-	HMDB0000318
UM15	4.82	459.1688	N-Acetyl-L-phenylalanine	↑	-	HMDB0000512
UM16	5.16	363.074	1-Methyluric acid	↑	-	HMDB0003099
UM17	5.55	248.0949	3-Indolebutyric acid	↓	-	HMDB0002096
UM18	5.68	429.1932	17-beta-Estradiol-3-glucuronide	↑	-	HMDB0006224
UM19	7.15	329.0695	Glycitein	↓	-	HMDB0005781
UM20	7.4	381.2268	Leukotriene B4	↑	-	HMDB0001085
UM21	7.57	453.1757	L-Tryptophan	↑	-	HMDB0000929
UM22	7.84	343.0839	Hydantoin-5-propionic acid	↓	-	HMDB0001212
UM23	9.73	397.1858	5-hydroxytryptamine	↑	-	HMDB0000259
UM24	10.73	407.2788	Cholic acid	↑	-	HMDB0000619

UM25	13.18	299.2007	all-trans-Retinoic acid	↑	-	HMDB0001852
UM26	13.52	299.2017	(R)-Carvone	↑	-	HMDB0035089
UM27	14.31	299.2589	2-Hydroxystearic acid	↑	-	HMDB0062549

a: PM: metabolites from plasma; UM: metabolites from urine.

b: The trend is model group vs control group: ↑, increase; ↓, decrease.

Table S4 Investigation of SCFA linear relationship

Compound	Linear equation	R2	Linear range（mg/ml）
AA	y=1389.5x+88051	0.9944	0.81~5.18
PA	y=758.7x+50944	0.996	0.76~4.85
IBA	y=1348.8x-199487	0.9916	0.72~4.59
BA	y=1028.6x+48224	0.9901	0.71~4.53
IVA	y=1094.5x+22249	0.9945	0.68~4.37
VA	y=741.44x+18782	0.9912	0.70~4.51

Table S5 Precision, repeatability, stability and recovery (n=6) of SCFA

Compound	Precision (RSD/%)	Repeatability (RSD/%)	Stability (RSD/%)	Recovery（%）	
				Mean	RSD
AA	1.32	2.14	3.09	98.38	1.91
PA	2.88	2.24	2.95	95.94	1.97
IBA	2.66	3.65	2.48	96.68	3.10
BA	2.69	2.37	2.16	100.80	1.20
IVA	2.85	2.46	3.47	97.63	2.85
VA	2.85	1.55	2.33	98.97	1.81

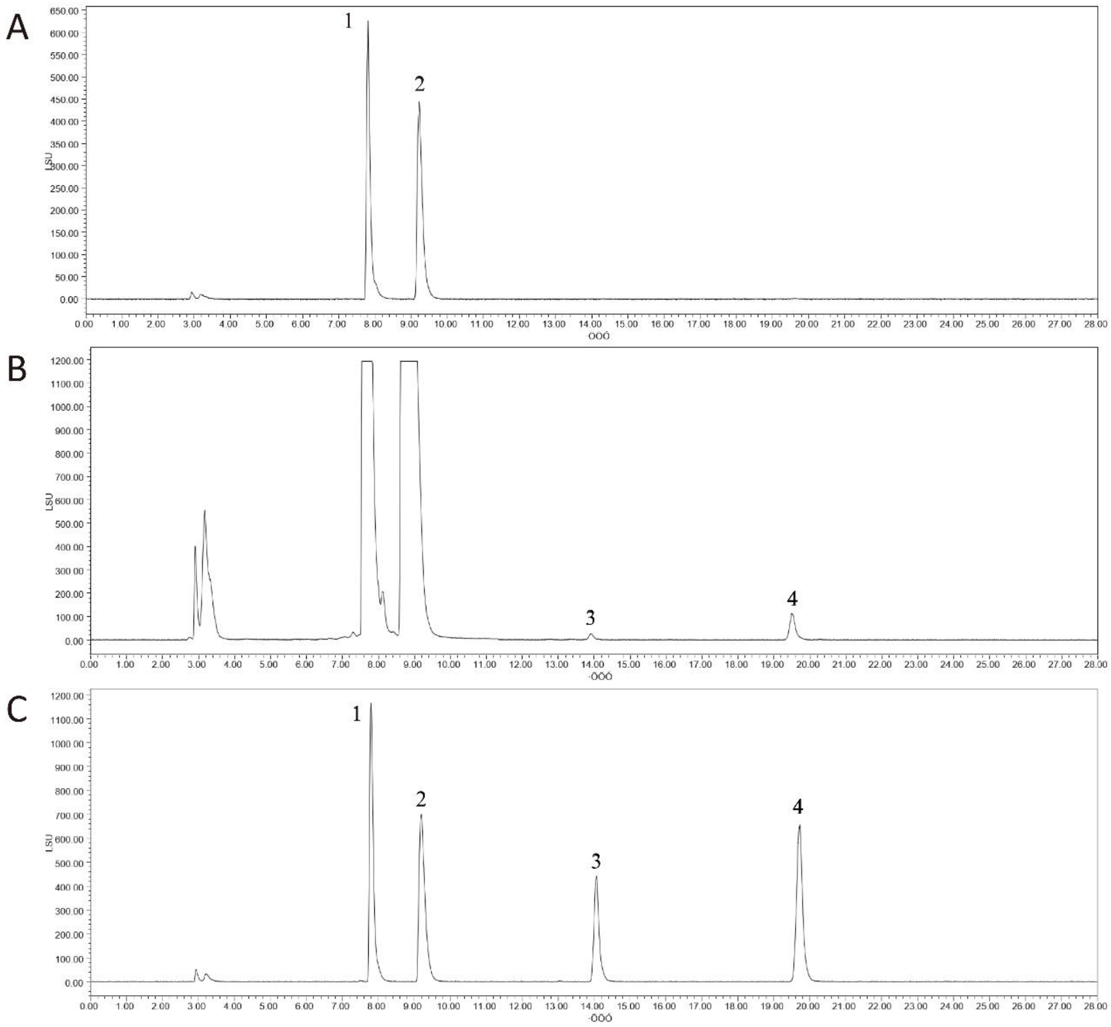

Figure S1 HPLC-ELSD chromatogram of BBP (A, B), and tauroursodeoxycholic acid (1), taurochenodeoxycholic acid (2), ursodeoxycholic acid (3), and chenodeoxycholic acid (4) references (C)

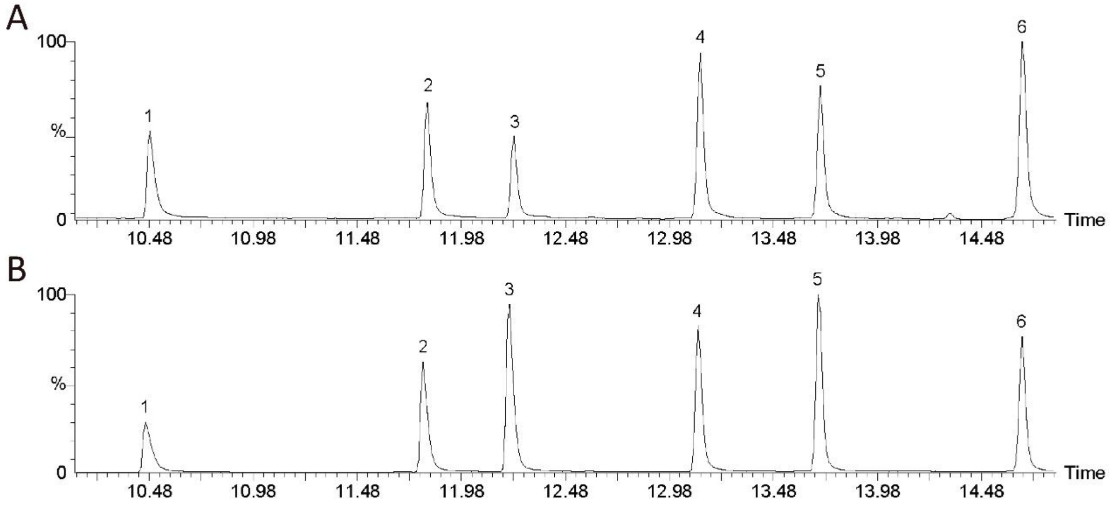

Figure S2 GC-FID chromatogram of short-chain fatty acids in feces (A), and acetic acid (1), propionic acid (2), isobutyric acid (3), butyric acid (4), isovaleric acid (5) and valeric acid (6) references (B)
